# The impact of financial incentives on participants’ food purchasing patterns in a supermarket-based randomized controlled trial

**DOI:** 10.1186/s12966-017-0573-0

**Published:** 2017-08-25

**Authors:** Dana Lee Olstad, David A Crawford, Gavin Abbott, Sarah A McNaughton, Ha ND Le, Cliona Ni Mhurchu, Christina Pollard, Kylie Ball

**Affiliations:** 1 Department of Community Health Sciences, University of Calgary, 3280 Hospital Drive NW, Calgary, T2N 4Z6 Canada; 2 Deakin University, Geelong, Australia, Institute for Physical Activity and Nutrition (IPAN), School of Exercise and Nutrition Sciences, Locked Bag 20000, Geelong, 3220 Australia; 3 Deakin University, Geelong, Australia, Deakin Health Economics, 221 Burwood Highway, Burwood, Victoria 3125 Australia; 4 National Institute for Health Innovation, School of Population Health, The University of Auckland, Private Bag 92019, Auckland Mail Centre, Auckland, 1142 New Zealand; 5 School of Public Health, Faculty of Sciences, Curtin University, GPO Box U1987, Perth, Western Australia 6845 Australia

**Keywords:** Supermarket, Food purchasing, Fruits and vegetables, Price reductions, Randomized controlled trial

## Abstract

**Background:**

The impacts of supermarket-based nutrition promotion interventions might be overestimated if participants shift their proportionate food purchasing away from their usual stores. This study quantified whether participants who received price discounts on fruits and vegetables (FV) in the Supermarket Healthy Eating for Life (SHELf) randomized controlled trial (RCT) shifted their FV purchasing into study supermarkets during the intervention period.

**Methods:**

Participants were 642 females randomly assigned to a 1) skill-building (*n* = 160), 2) price reduction (*n* = 161), 3) combined skill-building and price reduction (*n* = 160), or 4) control (*n* = 161) group. Participants self-reported the proportion of FV purchased in study supermarkets at baseline, 3- and 6-months post-intervention. Fisher’s exact and χ^2^ tests assessed differences among groups in the proportion of FV purchased in study supermarkets at each time point. Multinomial logistic regression assessed differences among groups in the change in proportionate FV purchasing over time.

**Results:**

Post-intervention, 49% of participants purchased ≥50% of their FV in study supermarkets. Compared to all other groups, the price reduction group was approximately twice as likely (RRR: 1.8-2.2) to have increased proportionate purchasing of FV in study supermarkets from baseline to post-intervention (*p*< 0.05).

**Conclusions:**

Participants who received price reductions on FV were approximately twice as likely to shift their FV purchasing from other stores into study supermarkets during the intervention period. Unless food purchasing data are available for all sources, differential changes in purchasing patterns can make it difficult to discern the true impacts of nutrition interventions.

**Trial registration:**

The SHELf trial is registered with Current Controlled Trials Registration ISRCTN39432901, Registered 30 June 2010, Retrospectively registered (http://www.isrctn.com/ISRCTN39432901).

## Introduction

Supermarkets represent an important environment for influencing food selection and consumption given that most of the household food budget is spent there, and their significant power and control over the global food supply [1]. This power can be readily leveraged to negative, or alternatively, to more health-promoting ends because of the vast array of both healthy and unhealthy foods supermarkets offer [2]. Food prices are a key driver of food purchasing behaviors [3, 4]. The relative prices charged by supermarkets for healthy and unhealthy foods may therefore be one means through which supermarkets influence food purchasing and consumption [5]. Indeed, increased availability of low-cost, ultra-processed, energy-dense foods may be one reason why diet quality has declined in recent decades [1, 6].

Given evidence that supermarket pricing influences food purchasing [4], several supermarket-based randomized controlled pricing interventions have been conducted, with collective findings suggesting that price discounts on targeted healthier foods can increase their purchase [7–11]. The Supermarket Healthy Eating for Life (SHELf) randomized controlled trial (RCT) examined the impact of 20% price reductions on fruits and vegetables (FV) and healthier beverages, skill-building, and a combined price reduction + skill-building intervention on food purchasing within a single supermarket chain [7]. Purchasing outcomes showed that in the immediate post-intervention period, the price reduction group increased purchasing of FV, while the combined group increased purchasing of fruit. However, findings from a process evaluation revealed that some participants randomized to receive price discounts reported having shifted their FV purchasing from other stores into study supermarkets to take advantage of discounts offered during the intervention period [12]. Previous supermarket-based RCTs have acknowledged that displacement of food purchasing might occur in pricing interventions [8], however to our knowledge no studies have confirmed that it does occur, nor examined its potential impact on study findings. Displacement of food purchasing is a concern because it may mask the true effectiveness of interventions. Thus, an apparent increase in FV purchasing within study supermarkets might actually represent a shift of FV purchasing from other food stores into study supermarkets, while overall FV purchasing remains unchanged. If similar shifts do not occur in the control group, change in food purchasing may be overestimated. The purpose of this study was to quantify whether, and to what extent participants who received price discounts on FV in the SHELf RCT may have shifted their FV purchasing from other stores into study supermarkets during the intervention period.

## Methods

### Participants and setting

Full methodological details of the SHELf RCT have been previously described [7, 13], and are briefly summarized here (Current Controlled Trials Registration ISRCTN39432901). The study was conducted amongst women living in one relatively advantaged, and one relatively disadvantaged neighborhood within Melbourne, Australia. These two neighborhoods were randomly selected from among those in the top (advantaged) and bottom (disadvantaged) tertiles of the Australian Bureau of Statistics’ Socioeconomic Index for Areas [14] that were serviced by a Coles® supermarket (the second largest grocery chain in Australia) and located within 25 km of Deakin University. Two stores from within these neighborhoods were purposively selected for the study, and women who shopped at least once every 2 weeks within either of these target stores or any other Coles supermarket within a 5 km radius comprised the sampling pool. A random sample of 3000 eligible women received a mailout of a study recruitment package to their home address. A media release targeting local newspapers was also undertaken in the catchment areas to encourage additional participation. To be eligible to participate women had to shop regularly within Coles supermarkets (at least once every 2 weeks), hold or be willing to obtain a Coles store loyalty (FlyBuys) card, be between 18 and 60 years of age, the main household food shopper, able to speak, read and write in English, willing to have their sales data collected and analyzed, and be the only woman in their household enrolled in the study.

The study was conducted between May, 2011 and November, 2012. All procedures involving human subjects were approved by the Deakin University Faculty of Health Human Ethics Advisory Group (approval HEAG-H 12/10) and adhered to the principles of the Helsinki Declaration. All women provided written, informed consent prior to participating.

### Intervention

Following completion of baseline surveys, participants were randomly allocated into one of four groups according to a computer-generated blocked randomization sequence produced and implemented by an independent statistician with a 1:1:1:1 participant allocation ratio: 1) Control (*n* = 161), 2) Price reductions (*n* = 161), 3) Skill-building (*n* = 160), or 4) Combined price reductions and skill-building (*n* = 160). Allocation concealment was ensured via the secure storage of the randomization sequence separately from the participant database, which was only accessible to the data manager and statistician. Women enrolled in the price reduction arm received 20% discounts on all FV (including all varieties of fresh, canned, dried and frozen FV, excluding fruit juice, frozen french Fries and fried potatoes), and on low-energy carbonated beverages and water. The skill-building group received a set of eight mailed skill-building newsletters including information, activities and recipes to promote purchase and consumption of FV and healthier beverages. Individuals in the combined group received 20% price discounts on FV and healthier beverages, along with all skill-building resources. The interventions took place over a 3-month period, with a further 6-month no-intervention follow-up phase. Women in the control group did not receive any interventions during the study period, and were asked to maintain their usual shopping habits.

### Data collection

Participants completed self-report surveys at baseline (T1), post-intervention (T2, 3 months) and 6-months post-intervention (T3, 9 months). At baseline, participants reported sociodemographic characteristics including highest educational qualification, marital status and the number of children living in the home. To assess proportionate food purchasing, at all three time points participants reported the estimated proportion of their total groceries (How much of your total food and groceries are bought from Coles supermarkets?) and of their FV (How much of your fruit and vegetables are bought from Coles supermarkets?) they purchased at Coles (response options: less than a quarter, between a quarter and a half, between half and three quarters, more than three quarters, all of them). Changes in proportionate food purchasing from T1 to T2, and from T2 to T3 were estimated on the basis of these responses. For example, participants who reported purchasing 25-50% of their FV at Coles at T1 and 50-75% of their FV at Coles at T2 were deemed to have increased proportionate FV purchasing at Coles from T1 to T2.

### Statistical analyses

Fisher’s exact (for small expected cell sizes) and χ^2^ tests assessed differences among intervention groups in self-reported proportionate food purchasing at each time point. Multinomial logistic regression with robust standard errors was used to assess differences among intervention groups in change (classified as: increase, decrease, no change) in self-reported proportionate food purchasing from T1 to T2 and from T2 to T3. Data from all participants who completed any portion of the T1 (*n* = 642; 100% of those randomized), T2 (*n* = 619; 96% of those randomized) or T3 (*n* = 606; 94% of those randomized) surveys were included in these analyses, thus the sample size differed for each analysis.

All statistical tests were two-sided and the significance level was set at *p* < 0.05. Stata software (version 13; StataCorp LP, TX) was used for all statistical analyses.

## Results

### Descriptive characteristics

The baseline sociodemographic characteristics of participants have been previously reported [7]. The sample was nearly evenly split according to neighborhood disadvantage, with 44% from low, and 55% from high socioeconomic status areas. Approximately half of the women were tertiary educated (50%) and had at least one child living at home (53%), and the majority were married (71%) [7]. On average, between 71 and 80% of participants reported purchasing more than half of their total groceries at Coles, whereas just 45-49% of participants reported purchasing more than half of their total FV at Coles at all three time points (Table 1). A minority reported purchasing more than 75% of their groceries (43-45%) and of their FV (25-28%) at Coles at all three time points.

**Table 1:** Proportionate food purchasing reported by participants in the SHELf RCT at baseline, post-intervention and 6-months post-intervention

	Baseline (%) *n* = 642				Post-intervention (%) *n* = 619				6-months post-intervention (%) *n* = 606			
Survey questions	Control *n* = 161	Price reduction *n* = 161	Skill-building *n* = 160	Combined *n* = 160	Control *n* = 157	Price reduction *n* = 158	Skill-building *n* = 151	Combined *n* = 153	Control *n* = 154	Price reduction *n* = 154	Skill-building *n* = 148	Combined *n* = 150
How much of your total food and groceries are bought from Coles supermarkets?												
All	4.4	5.0	6.9	5.6	5.1	3.8	6.6	7.9	2.6	7.1	7.4	8.0
75-99%	41.0	36.7	40.6	37.5	41.4	39.0	39.7	37.5	43.5	31.2	36.5	35.3
50-75%	33.5	32.9	35.0	40.6	27.4	35.9	28.5	32.9	26.0	27.9	27.0	31.3
25-50%	19.9	19.9	15.0	12.5	24.2	18.9	20.5	19.1	22.7	26.6	24.3	18.7
< 25%	1.2	5.6	2.5	3.8	1.9	2.5	4.6	2.6	5.2	7.1	4.7	6.7
How much of your fruit and vegetables are bought from Coles supermarkets?												
All	2.5	5.0	5.6	3.1	3.8	6.9	8.0	5.9	2.0	9.1	7.4	8.0
75-99%	27.3	18.0	21.3	19.4	22.9	22.0	21.9	19.7	24.0	15.6	17.6	14.7
50-75%	16.2	19.3	23.1	25.6	21.7	23.3	17.9	20.4	22.7	16.9	24.3	19.3
25-50%	29.2	26.1	25.0	20.6	24.8	25.8	23.8	25.0	23.4	27.3	21.6	24.0
< 25%	24.8	31.7	25.0	31.3	26.8	22.0	28.5	29.0	27.9	31.2	29.0	34.0

Fisher’s exact tests and χ^2^ tests assessed differences in survey responses among intervention groups at each time point. No differences were found

### Change in proportionate food purchasing

Up to 50% of participants reported changing their proportionate food purchasing in study supermarkets during the study period (Table 2). From T1 to T2, individuals in the price reduction group were significantly more likely to report having increased proportionate purchasing of FV at Coles compared to individuals in the control (RRR: 2.15; 95% CI: 1.26-3.68), skill-building (RRR: 1.94; 95% CI: 1.14-3.32) and combined (RRR: 1.82; 95% CI: 1.08-3.06) groups (*p* < 0.05). There were no other differences in change in proportionate food purchasing among groups from T1 to T2, or from T2 to T3.

**Table 2:** Proportion of participants in the SHELF RCT who reported changing their proportionate food purchasing in study supermarkets

	Change from baseline to post-intervention (%) *n* = 619				Change from post-intervention to 6-months post-intervention (%) *n* = 606			
	Control *n* = 157	Price reduction *n* = 158	Skill-building *n* = 151	Combined *n* = 153	Control *n* = 154	Price reduction *n* = 154	Skill- building *n* = 148	Combined *n* = 150
Change^a^ in proportion of total food and groceries bought from Coles								
Increase	18.5	23.9	18.5	21.7	19.7	19.0	19.7	19.1
No change	58.0	58.5	56.3	54.6	53.3	50.3	61.3	57.8
Decrease	23.6	17.6	25.2	23.7	27.0	30.7	19.0	23.1
Change^a^ in proportion of fruits and vegetables bought from Coles								
Increase	19.1	35.2	20.5	23.0	22.4	21.6	21.1	19.1
No change	58.0	49.7	56.3	59.2	55.3	43.1	53.5	57.1
Decrease	22.9	15.1	23.2	17.8	22.4	35.3	25.4	23.8

^a^Change in self-reported fruit and vegetable purchasing was calculated as the difference between the proportion of fruits and vegetables participants reported to have purchased in study supermarkets at T2 compared to T1, and in the proportion of fruits and vegetables participants reported to have purchased in study supermarkets at T3 compared to T2

## Discussion

Supermarkets represent an important environment in which to intervene to improve population-level dietary behaviors. RCTs provide high quality data in support of causal inference, however even RCTs have limitations, particularly in the context of complex, real-world, population-level nutrition interventions [15, 16]. In the SHELf RCT, individuals in the price reduction group were approximately twice as likely to report having increased proportionate purchasing of FV in study supermarkets from baseline to post-intervention, relative to all other groups. Qualitative comments from a prior process evaluation substantiated these findings, and suggested that these differential changes in FV purchasing were related to the financial incentive provided by the 20% price reductions [12]. SHELf is the first study, to our knowledge, to formally assess the patterning of proportionate food purchasing within the context of a supermarket-based RCT, albeit on the basis of self-reported data.

While RCTs are undoubtedly the gold standard of research design, collecting data pre- and post-intervention, without measuring what happens in between, risks misstating the true impact of interventions on outcomes [15]. The current analysis was motivated by responses provided during a prior process evaluation of the SHELf RCT [12], and supported those earlier findings. When food purchasing data are not available from all sources, *differential* changes in proportionate food purchasing between randomized groups, such as those documented here, can make it difficult to discern the true impacts of nutrition interventions. This is because it becomes unclear to what extent measured changes in food purchasing are real, or merely represent shifts in food purchasing from other stores into study-associated venues, while overall purchasing remains constant. Notably, the Healthy Incentives Pilot rebate program also found evidence to suggest participants randomized to receive rebates may have partially shifted their FV purchases into participating retailers to maximize rebate earnings [17].

The current findings appear to be the result of two factors operating in tandem. First, the intervention and comparison conditions were somewhat asymmetric in terms of their perceived benefits to participants. Participants who received price reductions had a strong financial incentive to displace their FV purchasing from other stores into study supermarkets, whereas those in the control and skill-building groups did not [12]. Second, purchasing data were only collected within Coles supermarkets, and proportionate FV purchasing was low at Coles at all three time points (i.e. less than half of participants reported purchasing ≥50% of their FV at Coles, with only one-quarter purchasing ≥75% of their FV at Coles at all three time points). It is likely to be the co-occurrence of these circumstances that proved problematic. For instance, had complete purchasing data for participants been available for all venues in which they shopped, any asymmetries in the nature of the intervention and comparison conditions would have been relatively immaterial. Conversely, had the price reduction groups not had a strong incentive to displace their food purchasing, our inability to measure purchasing in all venues should not have posed a substantial limitation because randomization should have ensured similar proportions of participants changed their food purchasing behaviors over time.

It is not clear why a similar displacement of proportionate FV purchasing into study supermarkets was not evident in the group that received a combination of price reductions and skill-building. This result is, however, consistent with the different food purchasing and consumption behaviors exhibited by the price reduction and combined groups throughout the SHELf study [7]. This finding additionally suggests that while differential displacement of food purchasing may occur in some supermarket-based interventions, it is not necessarily inevitable, and should therefore be quantified and not assumed. It is also unclear why the change in self-reported proportionate FV purchasing in the price reduction group was not reflected in a similar shift in this group’s total grocery purchasing, however we expect this may be because FV purchases comprised a relatively small proportion of participants’ total food purchasing (i.e. participants purchased on average just 2.6 kg/week of FV) [7]. Finally, although 35% of those in the price reduction group reduced FV purchasing at Coles from T2 to T3 compared to an average of 22-25% in other groups, this decline was not statistically significant.

### Study limitations and strengths

The current findings derive from self-reported data, however they are consistent with economic theories of human behavior, and are supported by regression analyses demonstrating directional correspondence between change in reported proportional, and objectively quantified FV purchasing (data not shown), as well as by qualitative comments provided during a prior process evaluation [12]. Moreover, identical questions were asked at all three time points, there was little missing data, and there is no reason to expect differential reporting of proportionate food purchasing over time.

### Implications

The challenges associated with quantifying intervention-associated change in food purchasing described herein may be difficult to address given that modern environments offer ubiquitous access to food, and that incentives to alter food purchasing behaviors are inherent within the design of some supermarket-based interventions. The degree to which these and other potential biases arise will likely vary across studies according to factors such as the specific nature of each intervention, study location, and the participant population. Given that economic incentives are increasingly being deployed in an attempt to improve lifestyle behaviors, the current findings may also have broader relevance to other types of studies.

It is not possible to quantify to what extent shifts in food purchasing affected outcomes in the SHELf study. However, correspondence between purchasing and intake measures within the price reduction group provides reassurance that study-associated increases in fruit purchasing were not solely the result of displacement of proportionate fruit purchasing into study supermarkets. Moreover, that measured fruit purchasing increased in the combined group, a group in which significant displacement of proportionate FV purchasing was not reported is also instructive, as are qualitative comments from participants indicating that price discounts led them to purchase more FV overall. Authors of similar supermarket-based price interventions have employed sensitivity analyses [8], statistical adjustment [10], triangulation of measures [10, 11], and participant pledges to shop exclusively within study supermarkets [11] to avoid potential biases associated with displacement of food purchasing. The effectiveness of such strategies remains uncertain, however.

Study findings suggest that measurement of total food purchasing may be essential. These data could be obtained by asking participants to scan the bar codes of packaged foods brought into the home (e.g. analogous to the Neilsen Homescan Panel [18]) and to photograph unpackaged items, or by supplementing electronic purchasing data from study supermarkets with receipts from foods purchased elsewhere. However, just as individuals underreport food intake, households may also underreport their supermarket shopping [19, 20]. These additional measures might also deter study participation due to increased participant burden, although in one study participants deemed collection of food receipts a useful tool in helping them to purchase healthier foods [21]. Development of new, technology-based integrated data collection methods may simultaneously improve the validity of the data collected, while reducing participant burden.

## Conclusions

Supermarket-based interventions are a high priority given their substantial potential to improve food purchasing and intake at a population-level. Rigorous research designs are essential to ensure high quality data. RCTs are rightly regarded as the gold standard of research design, nevertheless they are not without limitations, particularly in the context of complex, real-world, population-level nutrition interventions [15, 16]. In the SHELf RCT, compared to all other groups, participants who received price reductions on FV were approximately twice as likely to report having increased proportionate purchasing of FV in study supermarkets during the intervention period, potentially displacing a portion of their food purchasing from other venues during the intervention period. When food purchasing data are not available from all sources, differential changes in purchasing patterns such as those documented here can make it difficult to discern the true impacts of nutrition interventions. Future studies should attempt to proactively mitigate these potential biases, and where they do arise, should quantify their level and impact. Novel areas of inquiry could relate to development and testing of technology-based data collection methods, or to post-hoc strategies to mitigate the impact of such biases.

## Abbreviations

FV: fruits and vegetables
RCT: Randomized controlled trial
SHELf: Supermarket healthy eating for life
